# FlowerPower: clustering proteins into domain architecture classes for phylogenomic inference of protein function

**DOI:** 10.1186/1471-2148-7-S1-S12

**Published:** 2007-02-08

**Authors:** Nandini Krishnamurthy, Duncan Brown, Kimmen Sjölander

**Affiliations:** 1 Department of BioEngineering, 473 Evans Hall #1762, University of California, Berkeley, CA, 94720-1762, USA; 2 UC Berkeley and UCSF Joint Graduate Group in Bioengineering, University of California, CA, USA

## Abstract

**Background:**

Function prediction by transfer of annotation from the top database hit in a homology search has been shown to be prone to systematic error. Phylogenomic analysis reduces these errors by inferring protein function within the evolutionary context of the entire family. However, accuracy of function prediction for multi-domain proteins depends on all members having the same overall domain structure. By contrast, most common homolog detection methods are optimized for retrieving local homologs, and do not address this requirement.

**Results:**

We present FlowerPower, a novel clustering algorithm designed for the identification of global homologs as a precursor to structural phylogenomic analysis. Similar to methods such as PSIBLAST, FlowerPower employs an iterative approach to clustering sequences. However, rather than using a single HMM or profile to expand the cluster, FlowerPower identifies subfamilies using the SCI-PHY algorithm and then selects and aligns new homologs using subfamily hidden Markov models. FlowerPower is shown to outperform BLAST, PSI-BLAST and the UCSC SAM-Target 2K methods at discrimination between proteins in the same domain architecture class and those having different overall domain structures.

**Conclusion:**

Structural phylogenomic analysis enables biologists to avoid the systematic errors associated with annotation transfer; clustering sequences based on sharing the same domain architecture is a critical first step in this process. FlowerPower is shown to consistently identify homologous sequences having the same domain architecture as the query.

**Availability:**

FlowerPower is available as a webserver at .

## Introduction

Biological processes such as speciation, gene duplication, and domain shuffling produce families of related genes whose gene products can have vastly different molecular functions. Inference of protein function in these cases has been shown to be prone to systematic error [1-3]. Phylogenomic analysis – inferring the function of a protein in the larger context of a protein family based on evolutionary relationships – addresses these errors and improves the accuracy of functional classification [2,4]. In a phylogenomic approach, a phylogenetic tree is constructed from a multiple alignment of evolutionarily related sequences. The tree topology is analyzed to discriminate orthologs from paralogs, and is overlaid with existing experimental data for the members of the family. Functional inference can then be performed in an evolutionary context.

Protein domains are independently folding structural units that often confer specific functions. Roughly 65% of eukaryotic proteins and 40% of prokaryotic proteins are composed of multiple domains [5,6]. Domain fusion and fission events produce "families" of proteins that may share only a single domain in common, and some domains are "promiscuous," in that they are present in many different domain architectures. In automated functional inference approaches, these "local" (partial) homologs are often retrieved using database search, and may, in fact, be the top hits; they may also be placed as siblings in a phylogenetic tree with proteins having entirely different domain structures. Since the function of a multi-domain protein is a composite of all its constituent domains, annotation transfer based on local homology – even in a phylogenomic context – can be misleading. This issue has received less attention than differentiation of orthologs and paralogs, but is the source of a significant number of annotation errors.

The most commonly used methods for clustering homologous proteins are BLAST [7] and PSI-BLAST [8]. There are three primary problems with the use of these and similar tools in the context of phylogenomic inference of molecular function. First, these methods are optimized for homolog detection based on local similarity; clusters are not screened to remove proteins with different domain structure. Second, overly permissive parameterization of these tools – particularly iterative methods such as PSI-BLAST – can result in the inclusion of non-homologs. Third, it is possible for repeated iterations of the homolog identification process to result in *profile drift*, with the result that the seed sequence may not be included in the final cluster, or the profile may have drifted to include non-homologs in the set.

Structural phylogenomics combines evolutionary and structural analysis to elucidate changes in molecular function and structure in protein superfamilies. This approach has several applications, one of which is predicting the molecular function of unknown proteins in an evolutionary context. Phylogenomic inference has been shown to reduce the systematic errors associated with function prediction by homology; integration of structural information (or prediction) improves the accuracy of this approach. Our recommended protocol for protein function prediction integrates structural considerations in the first step of a phylogenomic pipeline, i.e. gathering homologs that share the same domain architecture. For this task we present FlowerPower, a method that discriminates between local and global homologs with much higher precision than BLAST, PSIBLAST and the UCSC SAM Target-2K (T2K) hidden Markov model (HMM) method [9]. We also present examples of sequence annotation errors detected through the use of structural phylogenomics, which could have been avoided at the outset by adopting this approach.

## Results and discussion

We compared FlowerPower, BLAST, PSI-BLAST and T2K on the task of discriminating between proteins sharing the same domain architecture (global homologs) and those having local similarity but different overall domain structures (local homologs). BLAST and PSI-BLAST are the most commonly used methods for clustering homologous sequences. The T2K method is less well known, but has been shown to outperform all other methods at remote homolog detection [10]. For these experiments, we selected nine sequences whose domain structures could be confidently predicted by PFAM [11]. Each method was allowed to select sequences from the SwissPFAM database [12]. Method parameters were varied to assess the impact on sensitivity (recall: fraction of global homologs selected) and precision (selectivity: fraction of selected sequences that were global homologs).

**Figure 1:**
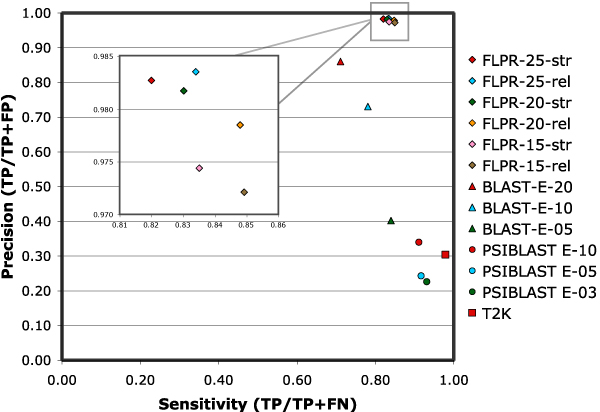
**Comparison of performances of BLAST, PSIBLAST, T2K and FlowerPower in identifying global homologs**. The X-axis refers to the average sensitivity (or recall) of each method across the dataset and the Y-axis refers to the average precision (or selectivity). Sensitivity is the fraction of the target homolog set identified by a method (i.e., TP/TP+FN). Precision is the fraction of the set selected by a method that belongs to the same domain architecture class (i.e., TP/TP+FP). Results of FlowerPower at varying parameterizations are presented including percent identity cutoffs for sequence selection (25%, 20% and 15%) and stringent ("str") and relaxed ("rel") query and hit coverage cutoffs, based on sequence length. The BLAST parameters refer to e-value cutoffs of 10^-20^, 10^-10 ^and 10^-5^. For PSI-BLAST the e-value cut-off used were 10^-10^, 10^-5 ^and 10^-3^, using three iterations. T2K was run using default parameters. The inset displays FlowerPower results using different parameterizations.

These experiments underscored the classic tradeoff between sensitivity (or recall) and precision: restrictive cutoffs yield the fewest false positives, improving precision, but reducing the recall rate. When inclusion cutoffs are relaxed, or iterated methods are used, recall improves, but precision often degrades, including many sequences with only partial (local) homology to the query. For instance, the SAM T2K method had the highest sensitivity, at 97.9%, but the worst selectivity, at 19.5% (i.e., eight out of ten sequences retrieved had different overall domain structures). PSI-BLAST, using E-value inclusion cutoffs of 10^-10^, 10^-5 ^and 10^-3^, had the next highest sensitivity (91.1%, 91.6% and 93.1% respectively) but poor precision (between 22.6% and 34%). A conservative parameterization of BLAST produced much higher precision than PSI-BLAST and T2K, but lower sensitivity: using an E-value cutoff of 10^-20 ^(the most restrictive cutoff tested), BLAST had a precision of 86%, but retrieved only 71.1% of global homologs. At the most permissive cutoff – an E-value of 10^-5 ^– BLAST precision dropped to 40% but retrieved 84% of global homologs. FlowerPower achieves the best precision of all methods tested – over 97%, regardless of method parameterization – and recovers between 82–85% of global homologs. Our recommended FlowerPower parameterization yields a recall of 85% and an average precision of 98%. See Figure 1.

The structural and functional variability in protein families complicates every aspect of an automated structural phylogenomic pipeline. Restricting a set of sequences to global homologs can be particularly challenging in these circumstances, as local similarity can result in sequences with different domain architectures being included in a dataset. These partial homologs can introduce errors in function prediction based on homology, as illustrated below.

### Examples of database annotation errors

We include here two examples of misannotated sequences which we discovered using structural phylogenomic inference of protein families in constructing our PhyloFacts phylogenomic resource [13]. The first sequence appears to have been annotated entirely by homology with a protein with strictly partial (local) similarity. The second, intriguingly, has been investigated experimentally, but neither the presumed species of origin (human) nor the assigned domain structure agree with that suggested by structural phylogenomic inference. While many annotation errors can also be detected through the use of domain structure analysis (e.g., through the use of PFAM or similar domain prediction webservers), the use of FlowerPower to cluster sequences sharing the same domain structure enables us to identify potentially erroneous annotations as anomalous in the context of the family as a whole.

#### Rice protein XP_478746

**Figure 2:**
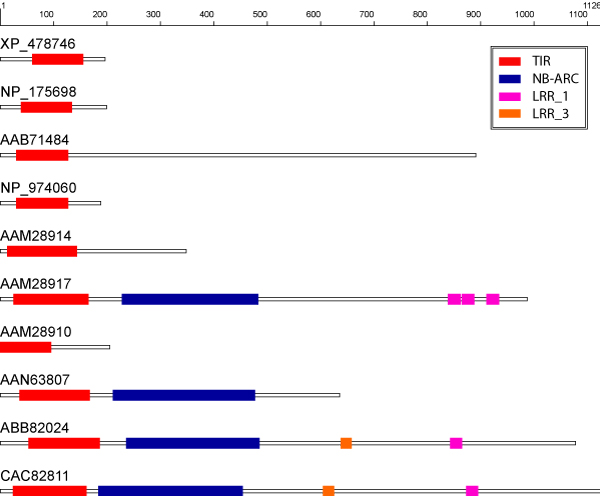
**Analysis of rice protein XP_478746 and BLAST hits**. The PFAM domain architectures of XP_478746, annotated as a "TIR/P-loop/LRR disease resistance protein-like protien" (sic) from *Oryza sativa*, and its closest BLAST homologs are shown. XP_478746 contains a TIR domain only with no room for the P-loop and LRR regions; the sequence is therefore misannotated. We expect XP_478746 was annotated based on local similarity to a sequence such as AAM28917 (annotated as a "putative TIR/NBS/LRR disease resistance protein" from *Pinus taeda*), which does contain the NB-ARC and LRR domains. See text for details.

*Oryza sativa *sequence XP_478746 is a 196-residue protein annotated as "TIR/P-loop/LRR disease resistance protein-like protein". Domain analysis using PFAM reveals that this protein contains only a TIR domain, which occupies almost the entire length of the sequence, leaving no room for either a P-loop or leucine-rich-repeat (LRR) region. We submitted this sequence to the BLAST server at NCBI, and analyzed the PFAM domain architecture of the top ten BLAST hits. Although each of the top ten BLAST hits had significant e-values (<10^-50^), the majority had domain architectures quite different from the query. Of the top ten BLAST hits, only four matched the query in containing a TIR domain only and being roughly the same length, four contained obviously different domain structures (including NB-ARC or NB-ARC/LRR domains), and two contained a TIR domain and long (almost 200aa and >700aa) undefined regions making them unlikely to share the same domain structure (see Figure 2). We expect the misannotation of XP_478746 was based on a database hit such as the 987-residue *Pinus taeda *sequence AAM28917 (one of the top BLAST hits), which contains a TIR domain, (P-loop containing) NB-ARC domain and LRR region.

FlowerPower, on the other hand, had much higher precision in the 43 sequences it selected from the NR database. PFAM analysis of FlowerPower sequences, requiring global matches to PFAM domains (*PFAM-ls*), finds only TIR domains in each of the sequences selected by FlowerPower. Allowing partial matches (*PFAM-fs*) detects fragmentary matches to other domains in two sequences: one (GenBank accession AAL07540) contains a fragmentary match to an NB-ARC domain, and the second (GenBank accession BAD94633) has a short (43aa) partial match to a motile sperm domain. SMART [14] detects only TIR domains in the FlowerPower sequences.

Details of these analyses are available in Supplementary Materials. A structural phylogenomic analysis of this protein, including the FlowerPower cluster, is available at [15].

#### Putative human neutral sphingomyelinase (AAF19052)

**Figure 3:**
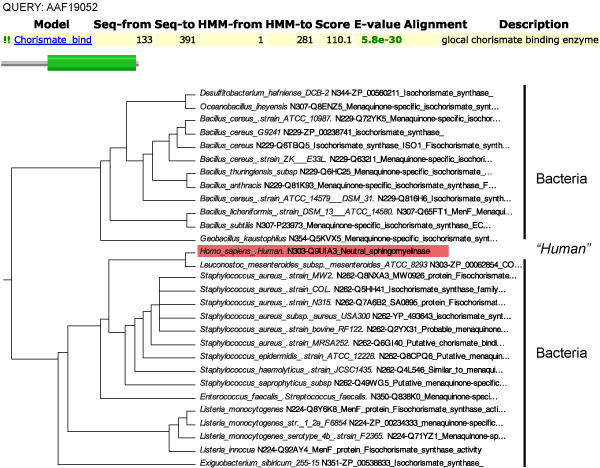
**Human sphingomyelinase or bacterial isochorismate synthase? **The sequence AAF19052 is reported to be a human neutral sphingomyelinase, containing a DEATH domain at the C-terminus. The top panel shows the PFAM domain architecture, which reveals the presence of a chorismate-binding domain at the C-terminus and the absence of a DEATH domain. The lower panel displays a structural phylogenomic analysis, resulting from clustering the sequence homologs to AAF19052 using FlowerPower and construction of a Maximum Parsimony tree. Examination of the phylogenetic tree suggests that AAF19052 (red box) is more likely an isochorismate synthase of bacterial origin. Each node in the tree is labeled with the species of origin, SCI-PHY subfamily label (see Methods), and sequence identifier and definition line.

AAF19052 is annotated as a human neutral sphingomyelinase, and is reported to contain a DEATH domain at the C-terminus [16]. Structural phylogenomic analysis shows all close homologs are from bacteria, and identifies a C-terminal chorismate-binding domain, but no DEATH domain. In addition, translated BLAST against the human genome finds no matches. Structural phylogenomic analysis for this protein, using FlowerPower to gather homologs, suggests that AAF19052 is more likely to be a bacterial isochorismate synthase; see [17]. See Figure 3.

## Conclusion

FlowerPower is designed for the first step in structural phylogenomic inference of protein function: selecting a dataset upon which functional inference will be based. For phylogenomic inference to be accurate, all sequences in the set must share the same domain architecture. FlowerPower has been shown to outperform BLAST, PSI-BLAST and the HMM-based SAM-T2K method at discriminating between proteins sharing the same domain structure and those having only local similarity. The precision of FlowerPower is much higher than the other methods tested, with an average false positive error rate under 3%, though PSI-BLAST and SAM-T2K have better sensitivity.

We have presented two examples of sequences with errors in their domain structure annotations producing errors in function prediction, which would have been prevented had a structural phylogenomic inference protocol been adopted. In these two cases, errors could also have been prevented through domain-structure analyses of each individual sequence using resources such as PFAM. FlowerPower provides an independent means of both preventing such errors and *post-hoc *identification of existing errors, through *anomaly detection*. Clustering sequences using a method such as FlowerPower enables us to assume all (or at least, most) of the sequences in the set have the same domain structure. If such a set contains sequences labelled differently, as in the examples presented here, oddball annotations will stand out as anomalous, signalling a potential error. Phylogenetic tree construction of these global homology clusters for phylogenomic inference of protein function enhances the specificity of functional annotation possible.

The FlowerPower method depends on two core methods to detect and align sequences: SCI-PHY subfamily identification and subfamily HMM construction. Subfamilies identified by SCI-PHY correspond closely to conserved clades found by phylogenetic analysis and to functional subtypes found by experts (*submitted*). Subfamily HMMs based on SCI-PHY subfamilies model the subtypes within a diverse protein family, accommodating lineage-specific structural and functional changes. Relative to the use of a single HMM for the family as a whole, subfamily HMMs have improved sensitivity at the same false positive rate: they identify dramatically more true positives under high significance cutoffs and provide greater separation between true and false positives. In addition, novel sequences can be classified to existing subfamily HMMs with very high accuracy [18].

A web server for FlowerPower is available at [19]. The default parameters are designed to retrieve sequences sharing the same domain structure; users can override the parameters to retrieve sequences sharing local or global-local similarity. Results include the native FlowerPower subfamily-HMM based alignment and a realignment of the sequences using MUSCLE. Users also have the option of generating functional subfamily using SCI-PHY and constructing subfamily HMMs. Alignments and SCI-PHY tree can be viewed online or downloaded.

### Future work

FlowerPower's high precision appears to be quite robust to different parameter settings, but there is clearly room for improvement in FlowerPower sensitivity. We plan to test the effect of other parameterizations of FlowerPower, including different ways to select the initial set for multiple sequence alignment, different SAM parameter settings for aligning sequences to subfamily HMMs, and score and alignment statistic requirements for inclusion of new sequences. Future experiments will also be performed on an expanded benchmark dataset, to ensure that method parameterization generalizes well to different protein architectures.

Profile drift is another area where we expect FlowerPower to provide superior performance. The use of subfamily hidden Markov models in retrieving new sequences in each iteration ensures that the seed and its homologs are included in the next iteration. Since subfamily HMMs improve the separation between true homologs and non-homologs, FlowerPower should prevent the intrusion of false positives in the final cluster. We plan to test FlowerPower's performance at these tasks.

## Methods

### The FlowerPower algorithm

FlowerPower is an iterative clustering algorithm akin to PSI-BLAST that retrieves and aligns sequences using profile methods. However, instead of using a single profile for homolog detection, FlowerPower constructs and uses subfamily HMMs to detect and align sequences in the next iteration; this is designed to improve alignment accuracy and prevent profile drift. Automated alignment analysis and quality control at every step ensure that homologs selected meet user-specified criteria. When parameterized for functional inference, quality control measures of alignment overlap and sequence identity result in clusters composed of sequences with the same domain architecture.

#### Selection of sequences for FlowerPower search

The input to FlowerPower is a seed sequence, a specified database from which putative homologs will be selected, and user-selected alignment quality control criteria (coverage and percent identity cutoffs). Due to the computational complexity of HMM scoring, the first step in FlowerPower involves running PSI-BLAST to gather a set of potential homologs against which subfamily HMMs will search. When FlowerPower is used to select globally alignable matches, this set is filtered to remove sequences that are clearly too long or too short to share the same domain structure as the seed. The final set, *S*, is then used as the target database from which potential homologs are selected using HMM scoring.

#### Creation of the initial multiple sequence alignment

We select a set of sequences with high sequence similarity to the seed, as follows. We construct an HMM for the seed sequence using the SAM modelfromalign software. Sequences from *S *with a BLAST E-value <= 10^-10 ^to the seed are aligned to the HMM using the align2model software; using HMMs to align the sequence produces somewhat more global alignments than produced by BLAST. Sequence alignments are then examined; sequences having >=25% pairwise identity to the seed and passing minimum fractional (length-dependent) bi-directional overlap between the database hit and the seed (termed the "coverage") are accepted. The "coverage" fraction varies between 0.60 for sequences of <100aa and 0.85 for sequences of >500aa. We then use MUSCLE [20] to realign the sequences. The MUSCLE alignment is submitted to SCI-PHY [21] for subfamily identification and subfamily HMM construction [18].

#### Iterated sequence retrieval and alignment

**Figure 4:**
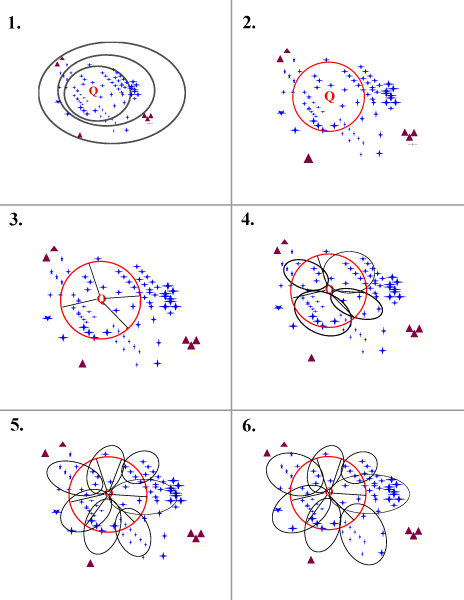
**The FlowerPower algorithm**. "Q" indicates the query (or seed) sequence. Sequences sharing the same domain structure are indicated as blue stars; all other sequences are indicated as brown triangles. SCI-PHY subfamilies are indicated by black ovals. **1**. Identify a set of potential homologs *S *using PSI-BLAST; filter to remove much longer or much shorter sequences. **2**. Select a core set for initial alignment. **3**. Identify subfamilies using SCI-PHY and construct subfamily HMMs (SHMMs). **4**. Score *S *with the SHMMs, and identify those sequences receiving scores with E-values below cutoff. Align each sequence to its closest SHMM. Evaluate the alignment with user-specified criteria; remove sequences that do not meet these criteria. **5**. Run SCI-PHY on the new alignment to identify subfamilies and construct SHMMs. **6**. Repeat steps 1–5 until convergence.

In subsequent stages, the sequences in *S *are scored with the subfamily HMMs using the SAM hmmscore program, selecting sequences for alignment based on score significance. At program commencement, the E-value cutoff for inclusion is fairly stringent (E-value <= 10^-10^) and is gradually increased (made less stringent) in each iteration to reach a maximum of 10^-5^. E-values are computed based on local-local scoring (SAM parameter - sw 2) and an assumed database size of 100,000. Each sequence receiving a significant score is aligned to the subfamily HMM giving it the strongest score, to produce a multiple sequence alignment of all sequences passing the E-value cutoff. Alignment quality control for minimum percent identity and bi-directional coverage is then performed to remove sequences not meeting these criteria (these values are user-specifiable parameters of the algorithm). The new multiple sequence alignment is provided as input to SCI-PHY, subfamily HMMs are constructed, and the process is iterated until no new sequences match inclusion criteria, or the number of sequences or iterations reaches a pre-defined cutoff. Additional details on alignment quality control parameters, including coverage and percent identity, are available in Supplementary Materials. See Figure 4.

### Subfamily identification using SCI-PHY

FlowerPower uses the SCI-PHY (Subfamily Classification In Phylogenomics) method to predict functional subfamilies in each iteration, based on the multiple sequence alignment of sequences retrieved at that stage. SCI-PHY constructs a hierarchical tree using agglomerative clustering, and cuts the tree into subtrees using a combination of information theoretic methods and Dirichlet mixture densities [22]. SCI-PHY uses an encoding cost measurement under a Dirichlet mixture density to determine an optimal cut of the tree into subtrees. SCI-PHY subfamily classification has been shown to correspond closely to phylogenetic clades and expert identified subtypes (*submitted*). A detailed description of the algorithm is published in [21].

### Subfamily HMM construction

Subfamilies identified by SCI-PHY are used to construct subfamily hidden Markov models, which are used to score and align sequences for the next iteration of FlowerPower. Subfamily HMM parameters are estimated using an information-sharing protocol enabling statistics to be shared across subfamilies in a position- and subfamily-specific manner. Amino acid distributions at positions conserved across the family are fixed for each subfamily; this ensures that even very small subfamilies include information about positions defining the family as a whole. At other positions, subfamilies share statistics with subfamilies aligning similar residues, while keeping their statistics separate from subfamilies aligning very dissimilar residues. This protocol retains specificity at subfamily-defining regions or motifs, while generalizing well to more distant homologs. This provides high specificity of classification while simultaneously improving the sensitivity of the subfamily HMM to detect new members [18]. Recent experiments on a large representative dataset of 515 SCOP folds show that subfamily HMMs dramatically increase the separation between true homologs and non-homologous proteins with different folds (*submitted*).

### Comparison of FlowerPower with BLAST, PSI-BLAST and T2K

BLAST was tested using three different e-value cut-offs: 10^-20^, 10^-10 ^and 10^-5^. For PSI-BLAST, we varied the number of iterations (three and five) and e-value cut-offs (10^-10^, 10^-5 ^and 10^-3^). The results from five iterations were almost indistinguishable to results obtained from using three iterations and are not shown. SAM-T2K was run using default parameters.

**Figure 5:**
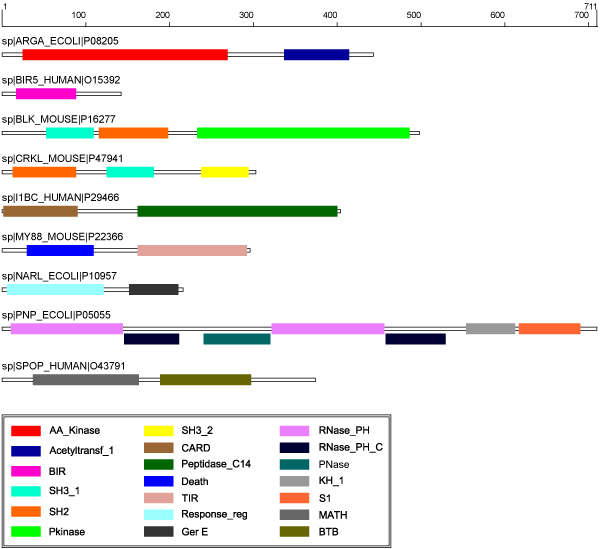
**PFAM domain architecture of the seed dataset used to evaluate BLAST, PSI-BLAST, T2K and FlowerPower**. Sequences were selected based on the following criteria: each sequence had to contain recognizable PFAM domains (based on the PFAM gathering threshold), no undefined regions of >80 amino acids (i.e., a region with no PFAM match), and each PFAM domain was required to match a 3D structure classified by the SCOP database. For details of the seed sequence domain architectures see Table 1.

Nine seed sequences for database search were selected from SwissPFAM [12] based on the following criteria: (1) a majority of the sequence matching PFAM domains, based on the PFAM gathering threshold, (2) no undefined regions of >80 amino acids (i.e., a region with no PFAM match), and (3) each PFAM domain matched a solved protein structure classified by the Structural Classification of Proteins (SCOP) database [23]. Table 1 and Figure 5 provide details of seed sequences used in these experiments.

BLAST, PSI-BLAST, T2K and FlowerPower were then used to retrieve proteins from SwissPFAM version 15. Retrieved sequences were labelled as *homologous*, *non-homologous*, or *indeterminate*. To be called *homologous*, a database hit had to be clearly in the same domain architecture class as the seed sequence (i.e., the same or structurally equivalent PFAM domains (based on SCOP analysis) in the same order), with any unlabelled region restricted to less than 80 amino acids. PFAM domains are considered equivalent if they match at the level of SCOP superfamily. For instance, the PFAM CARD, DEATH and DED domains are all members of the SCOP DEATH domain superfamily, and would be considered structurally equivalent in our analysis. The set of proteins in SwissPFAM matching these criteria form the full set of global homologs; methods identifying all the global homologs would therefore have perfect sensitivity. Proteins were defined as *non-homologs *if they were much longer than the seed (i.e., hit length > seed length + 500), contained a PFAM domain not homologous to any domain in the seed (based on disagreement at the level of SCOP fold), contained an unequal number of homologous PFAM domains, or had a different ordering of PFAM domains than those in the seed. All other proteins were called *indeterminate*, as their global structural homology or lack thereof could not be rigorously determined on the basis of these analyses. For a given homolog-detection method, we then define True Positive hits (TP, global homologs correctly selected by that method), True Negatives (TN, non-global-homologs that are correctly rejected), False Positives (FP, non-global-homologs that are incorrectly accepted), and False Negatives (FN, true global homologs that are incorrectly rejected). Results of these experiments are shown in Figure 1.

#### Phylogenetic tree construction and display

The phylogenetic tree shown in Figure 3 was estimated using Maximum Parsimony from the PAUP* software [24], and displayed using the ATV software [25].

## Accession numbers

[GenBank:XP_478746, GenBank:CAC82811, GenBank:ABB82024, GenBank:AAN63807, GenBank:AAM28910, GenBank:AAM28917, GenBank:AAM28914, GenBank:NP_974060, GenBank:AAB71484, GenBank:NP_175698, GenBank:AAM28917, GenBank:AAL07540, GenBank:BAD94633, GenBank:AAF19052, SwissProt:ARGA_ECOLI, SwissProt:BIR5_HUMAN, SwissProt:BLK_MOUSE, SwissProt:CRKL_MOUSE, SwissProt:I1BC_HUMAN, SwissProt:MY88_MOUSE, SwissProt:NARL_ECOLI, SwissProt:PNP_ECOLI, SwissProt:SPOP_HUMAN]

## Abbreviations

FN: False Negative, FP: False Positive, HMM: Hidden Markov model, SCOP: Structural Classification of Proteins, T2K: SAM Target2K, TN: True Negative, TP: True Positive

## Authors' contributions

KS conceived the work. NK and DB developed the required software, created the dataset and evaluated the methods. KS, NK and DB wrote the paper and created the figures.

## Figures and Tables

**Table 1:** Details of FlowerPower validation dataset.

**Seed ID**	**Seed length**	**Domain Architecture**	**Total GH**	**Total NH**	**Total IND**
ARGA_ECOLI	443	AA_kinase (26–269); Acetyltransf_1 (338–414)	26	898420	3559
BIR5_HUMAN	142	BIR (18–88)	23	901954	28
BLK_MOUSE	498	SH3_1 (54–109); SH2 (117–198); Pkinase (234–486)	76	893470	8459
CRKL_MOUSE	303	SH2 (14–88); SH3_1 (126–181); SH3_2 (239–294)	15	900640	1350
I1BC_HUMAN	404	CARD (2–91); Peptidase_C14 (163–401)	33	901717	255
MY88_MOUSE	296	Death (31–109); TIR(163–292)	4	897880	4121
NARL_ECOLI	216	Response_reg (7–128); GerE (153–210)	1757	884810	15438
PNP_ECOLI	711	RNase_PH (12–144); RNase_PH_C (147–211); PNPase (242–320); RNase_PH (323–456); RNase_PH_C (459–529); KH_1 (555–612); S1 (618–690)	121	885592	16292
SPOP_HUMAN	374	MATH (38–163); BTB (190–297)	68	901237	700

SwissProt identifiers are shown in the first column. The number of global homologs (GH; proteins sharing the same domain structure) for a seed was determined by the total number of proteins in the SwissPFAM dataset that shared the same domain structure as the seed. Non-homologs (NH) are those having an obviously different domain structure. Indeterminate sequences (IND) are those whose global homology to the seed or lack thereof could not be rigorously determined. See Methods for details.
